# Editorial: Biometric monitoring of emotions and behaviors

**DOI:** 10.3389/fnbot.2022.1079761

**Published:** 2022-11-16

**Authors:** Antonio M. López, Enrique León, Olga L. Quintero Montoya

**Affiliations:** ^1^Multisensor Systems and Robotics Laboratory (SiMuR), Electrical Engineering Department, University of Oviedo, Gijón, Spain; ^2^Departamento de Investigación Clínica, Corporación para Investigaciones Biológicas-CIB, Medellín, Colombia; ^3^Grupo de Investigación en Modelado Matemático, HuMath, Area de Computación y Analítica, Universidad EAFIT, Medellín, Colombia

**Keywords:** biosignal processing, biometric monitoring, behavior and emotion recognition, human-aware navigation and affective interaction, human-machine/robot interaction

In recent years new findings about how emotions influence our conduct have motivated a renewed attention from the public, the government, and the industry into the phenomena linked to affective interaction and individual emotions. The consequence of this growing interest has been that emotions have become a crucial element in the human-centered strategies of companies across the globe which in turn has propelled the market of affective computing applications. It is in this context that the monitoring of bodily signals becomes crucial since human behavior and decision making are heavily influenced by affective states which in turn are rooted in biology.

The seamlessly collection of biological, physiological, and behavioral data from humans is rapidly becoming a popular solution thanks to the ubiquitous deployment of environmental and wearable sensing devices. Biometric monitoring refers to the “conscious and unconscious changes of human traits and body parameters for assessing complex features such as emotions and behavior.” This has created new opportunities in fields such as security, fintech, health, educational technology and even construction and manufacturing. For instance, biometric monitoring will enable accurate identification of people in their interaction with computing systems and will also represent a revolution in the field of health where dense, continuous measurement of parameters of interest will be possible (as opposed to the measurements at specific points in time that have been performed so far).

Numerous use cases emerge from the utilization of biometric monitoring. A variety of measurable body variables such as electro dermal activity, heart rate response and eye movement or pupil dilatation can be used to estimate the emotional state and arousal of the subject. Likewise, gaze, head orientation or anticipatory postural adjustments provide information regarding the intended direction of movement, intention, or attention. Other data sources such as kinematic and kinetic parameters of body segments define the individual's motor activities.

It is therefore expected that biometric monitoring will drive breakthrough innovations that contribute to the industrial, environmental, and social revolution that digital technologies are supporting.

From this perspective, this Research Topic aims at contributing to an understanding of the importance of biometric monitoring for the modeling of human actions and behavior. Our goal is to foster the development of artificial cooperative applications that interact with humans in novel “behavioral” and “emotional” dimensions that employ biosensing systems. The works presented here were carefully selected to provide a broad and rich overview of the latest approaches in the field.

Some of the authors focus on fields that answer fundamental research questions. For example, Jirak et al. work at the emotional dimension, showing that it is possible to analyze human emotions from gaze behavior, arousal-valence and action units during the interaction of a person with a robot. Lim et al. present a systematic literature review from studies and articles relevant for eye-tracking classification over the last 5 years. From the analyzed data they address the definition of features for eye-tracking classification for machine learning procedures.

The work described in Díaz Leon and Quintero relates to the application dimension in physical and emotional rehabilitation. They extend the concept of Human centered Mathematics and develops the basic architecture of the developments in physical and emotional rehabilitation, prioritization and support for decision making using highly complex images.

Sarasola-Sanz et al. describe a complete materialization of the use of biometric monitoring to establish a cooperative system at the behavioral dimension. Departing from the low-level development of a myoelectric interface they successfully implemented the control of an exoeskeleton limb, showing promising findings for the rehabilitation of after-stroke patients.

Understanding how the brain works allows those of us who design new and better artificial intelligence algorithms to mathematically describe processes such as intelligence, learning, generalization, bias and other natural processes. As well as it allows to study the patterns in the physiological signals that are measured on the subject in the framework of an appropriate clinical study, they can be used for the development of artifacts. However, and with the advent of XR as a modifier of states of consciousness associated with emotion, it is possible to identify methodologies to allow the identification of “instances of emotion” from the initiation of various types of signal patterns by fusion of data. This capacity is especially valued in cyber-physical environments such as those of rehabilitation and even more so when the feedback from the subject is not purely mechanical, but rather the emotional awareness is a fundamental part of the process and must be included in the control loop.

The contents of the present Research Topic illustrate current guidelines and practical developments in the field of Biometric Monitoring of Emotions and Behaviors. The editorial board wishes to thank the contributors who share the goal of creating technology that enhances quality of life.

## Author contributions

All authors listed have made a substantial, direct, and intellectual contribution to the work and approved it for publication.

## Conflict of interest

The authors declare that the research was conducted in the absence of any commercial or financial relationships that could be construed as a potential conflict of interest.

## Publisher's note

All claims expressed in this article are solely those of the authors and do not necessarily represent those of their affiliated organizations, or those of the publisher, the editors and the reviewers. Any product that may be evaluated in this article, or claim that may be made by its manufacturer, is not guaranteed or endorsed by the publisher.

